# Epigenetic changes in fibroblasts drive cancer metabolism and differentiation

**DOI:** 10.1530/ERC-19-0347

**Published:** 2019-10-17

**Authors:** Rajeev Mishra, Subhash Haldar, Surabhi Suchanti, Neil A Bhowmick

**Affiliations:** 1 Department of Biosciences, Manipal University Jaipur, Jaipur, Rajasthan, India; 2 Department of Biotechnology, Brainware University, Kolkata, India; 3 Department of Medicine, Cedars-Sinai Medical Center, Los Angeles, California, USA; 4 Department of Research, Greater Los Angeles Veterans Administration, Los Angeles, California, USA

**Keywords:** endocrine therapy resistance, prostate, neuroendocrine tumors

## Abstract

Genomic changes that drive cancer initiation and progression contribute to the co-evolution of the adjacent stroma. The nature of the stromal reprogramming involves differential DNA methylation patterns and levels that change in response to the tumor and systemic therapeutic intervention. Epigenetic reprogramming in carcinoma-associated fibroblasts are robust biomarkers for cancer progression and have a transcriptional impact that support cancer epithelial progression in a paracrine manner. For prostate cancer, promoter hypermethylation and silencing of the RasGAP, RASAL3 that resulted in the activation of Ras signaling in carcinoma-associated fibroblasts. Stromal Ras activity initiated a process of macropinocytosis that provided prostate cancer epithelia with abundant glutamine for metabolic conversion to fuel its proliferation and a signal to transdifferentiate into a neuroendocrine phenotype. This epigenetic oncogenic metabolic/signaling axis seemed to be further potentiated by androgen receptor signaling antagonists and contributed to therapeutic resistance. Intervention of stromal signaling may complement conventional therapies targeting the cancer cell.

## Chromatin modification in cancer: a brief overview

Cancer is a general term for a group of diseases that diverge with respect to its origin and is characterized by uncontrolled proliferation with the potential for metastatic progression (Hanahan & Weinberg 2000, Chaffer & Weinberg 2011, Hanahan & Weinberg 2011). Cell proliferation is normally tightly regulated at the gene level with dynamic chromatin modifications (Perino & Veenstra 2016). Chromatin structure is central for the regulation of gene expression either by organizing the structure of promoters and regulatory elements or by providing accessibility to transcription factor binding at regulatory elements (Tirosh & Barkai 2008). One of the prime epigenetic phenomena in cancer is suppression or downregulation of tumor suppressor genes through aberrant promoter methylation and deacetylation, often associated with condensing the chromatin structure and preventing transcription factor loading, resulting in gene silencing (Robertson 2001, Luczak & Jagodzinski 2006). Conversely, the acetylation and demethylation of the gene-body can also result in gene silencing. The epigenetic activation of oncogenes on the other hand seem to be less associated with direct DNA or histone methylation/acetylation of the oncogenes themselves, but rather miRNAs that can indirectly regulate tumorigenic potential (Zhang *et al.* 2007, Mi *et al.* 2010, Yan *et al.* 2015). The role of miRNAs in the microenvironment is not discussed in this review as they are well reviewed elsewhere (Rupaimoole *et al.* 2016, Smith *et al.* 2017). However, the regulation of oncogene activity regulatory proteins of, as opposed to direct oncogene/tumor suppressor expression, can also result from DNA/histone modification. The tight oncogenic regulation suggests multiple mechanisms by which they can be subverted in the events leading to cancer.

The addition of a methyl group (CH_3_) at fifth carbon position of the cytosine ring of DNA, termed, 5-methylcytosine (5mC), predominantly occurs in CpG-rich sequences. Somatic, non-stem cells, normally have hypomethylated CpG islands in promoter sequences (Moore *et al.* 2013). However, aberrant promoter hypermethylation of multiple tumor-suppressor genes is associated with the upregulation of DNA methyltransferases (DNMTs) in multiple cancer types (Jin & Robertson 2013, Moore *et al.* 2013). The DNMT family comprises four members which include DNMT1, DNMT3A, DNMT3B and DNMT3L. All members of the family possess inherent enzyme activity except DNMT3L (Jin & Robertson 2013). While DNMT1 functions during DNA replication to maintain the DNA methylation pattern from the parental DNA strand onto the newly synthesized daughter strand, DNMT3a and DNMT3b are responsible for establishing *de novo* methylation pattern to unmodified DNA (Okano *et al.* 1998, 1999, Riggs & Xiong 2004, Egger *et al.* 2006, Goll *et al.* 2006). Epigenetic cancer therapeutic targets DNA/histone methylation in order to reverse chromatin remodeling (Sproul & Meehan 2013). An feature of cancer cell is the reduced total global DNA methylation in the context of enriched DNA methylation at certain promoter CpG islands (Wu *et al.* 2018). Laird *et al*. showed that heterozygotic mice with null mutation of *Dnmt1*, when treated with specific inhibitors of DNA methylation, such as 5-aza-2′-deoxycytidine (5-aza-dC) significantly reduced tumor formation in Apc Min^+/^^−^ mice (Takebayashi *et al.* 2007). Additional studies with gene knockout analysis in mice have shown that, a Dnmt1 hypomorphic allele (causing partial loss of function) can suppress polyp formation and CpG island methylation (Eads *et al.* 2002). In particular, studies have demonstrated that DNMT1 overexpression correlates with colon tumors, compared to non-malignant adjacent stroma (Honeywell *et al.* 2018). DNA methylation marks also involve active demethylation of 5mC by oxidizing enzymes including the ten-eleven translocation (TET) enzymes (TET1, TET2, TET3) as well as associated histone proteins by demethylase KDM4A/JHDM2A. Interestingly, epigenetic regulation can itself be regulated by metabolic intermediates. For example, the TCA cycle metabolite α-ketoglutarate is an inducer of TET2 (Raffel *et al.* 2017). The subsequent downstream metabolites, succinate and fumarate, promoted histone demethylation by KDM4A/JHDM2A (Xiao *et al.* 2012). New findings on the relationship between chromatin modification and cancer metabolism provide new opportunities for epigenetic therapy.

## Epigenetic coevolution of stromal fibroblastic cells in response to tumorigenesis

It is now established that carcinogenesis involves reciprocal interactions between cancer cells and components of the surrounding microenvironment consisting of extracellular matrix, fibroblasts, vasculature-associated endothelia and pericytes, as well as immune cells and occasionally adipose cells (Plava *et al.* 2019). Based on the pro-tumorigenic role these non-tumorigenic components have, tumor microenvironment-targeted interventions have attracted notable attention in cancer therapy (Dey 2011, Quail & Joyce 2017). Prominently, angiogenesis inhibitors have been practice-changing for a few cancer types, but interestingly had a lesser impact on cancer care than originally anticipated. Regulators of fibrosis have had limited efficacy. While immune therapy targeting T cell activation has taken cancer care by storm recently, thus far under 20% of melanoma and lung cancer patients demonstrate lasting benefit. Interestingly, there is a distinct change in the chromatin-accessible regions of exhausted T cells that is not alterable by immune checkpoint inhibition (Pauken *et al.* 2016, Sen *et al.* 2016). The understanding of the most abundant cell type of the solid tumor microenvironment, the fibroblasts, remains largely unknown. Not without controversy, cancer-associated fibroblasts (CAF), is considered not to be driven by genomic mutations (Hill *et al.* 2005, Li *et al.* 2007, Qiu *et al.* 2008, Bianchi-Frias *et al.* 2016). However, the seminal finding by Cunha and colleagues that CAFs have the capacity to maintain its tumor-inductive capacity in the absence of the constant signals from cancer cells for a period of time, suggested an inherent ‘memory’ (Olumi *et al.* 1999, Hayward *et al.* 2001). As evidence, CAF can be isolated from patient tissues, cultured, and then transferred to mice with non-tumorigenic cells to develop a tumor. In the absence of mutations, the pro-tumorigenic phenotype of CAF is found to be driven by epigenetic mechanisms associated with promoter DNA methylation (Dumont *et al.* 2008, Gascard & Tlsty 2016).

CAFs are the dominant cell type in tumor microenvironment, with both pro- and anti-tumorigenic capacity (Placencio *et al.* 2008, Kalluri 2016, LeBleu & Kalluri 2018). The net effect of paracrine signaling crosstalk between CAFs and the cancer epithelia provides avenues for disrupting pro-tumorigenic signaling (Wu *et al.* 2012). In contrast to normal tissue-associated fibroblasts (NAFs), the epigenetic programming in CAFs represents a durable change that is able to promote tumor growth (Fiori *et al.* 2019). The distinct contribution of TME epigenetic landscapes in tumorigenesis was first highlighted by Hu and colleagues (Hu *et al.* 2005) by developing a novel method – methylation-specific digital karyotyping tissue obtained from epithelial and stromal fibroblasts from normal breast and *in situ* and invasive breast carcinomas. This study highlighted that epigenetic landscape has a role in the maintenance of the abnormal microenvironment in breast cancer. In prostate cancer, pi-class glutathione S-transferase gene (*GSTP1*) promoter is methylated in >90% cases (Lee *et al.* 1994). This seminal study demonstrated distinct *GSTP1* gene promoter methylation of the stromal cells in prostate cancer. Although the primary role of GSTP1 is in the detoxification of carcinogens (Allocati *et al.* 2018), it is not involved in the suppression of cancer cell growth and cannot be classified as a tumor suppressor gene (TSG); however, its aberrant silencing in CAFs may create a permissive microenvironment for tumorigenesis (Lee 2007). In agreement Rodriguez-Canales *et al.* demonstrated significant topographical differences and distinct area of stromal methylation of the stroma especially at the center of the tumor in the prostate using laser capture microdissection (Rodriguez-Canales *et al.* 2007). We have reported that the epigenetic silencing of the TGF-β type II receptor (*Tgfbr2*) in prostatic CAF can be causative for GSTP1 promoter methylation, as the knockout of the *Tgfbr2* resulted in GSTP1 silencing in addition to a number of DNA damage repair genes (Banerjee *et al.* 2014). In addition, prostatic human CAF and mouse transgenic knockout of *Tgfbr2* demonstrated elevated DNA methyltransferases I (DNMT1) activity and histone H3 lysine 9 trimethylation (H3K9me3) associated with greater promoter methylation. Notably, restoring the expression of the epigenetically silenced genes in the CAF using 5-azacitidine led to reduced tumor progression (Banerjee *et al.* 2014). Promoter DNA and histone methylation can mediate a tumor permissive environment (El-Osta & Wolffe 2000, Rose & Klose 2014). A recent study showed that CAFs with a large number of H3K27me3 changes had greater tumor-promoting effects, associated with the secretion of the paracrine factor WNT5a (Maeda *et al.* 2019). The epigenetic landscape of PCa CAF has diagnostic and grading capacity of PCa (Gordetsky & Epstein 2016, Pidsley *et al.* 2018).

## DNA methylation and histone modification studies in CAF

**Figure 1:**
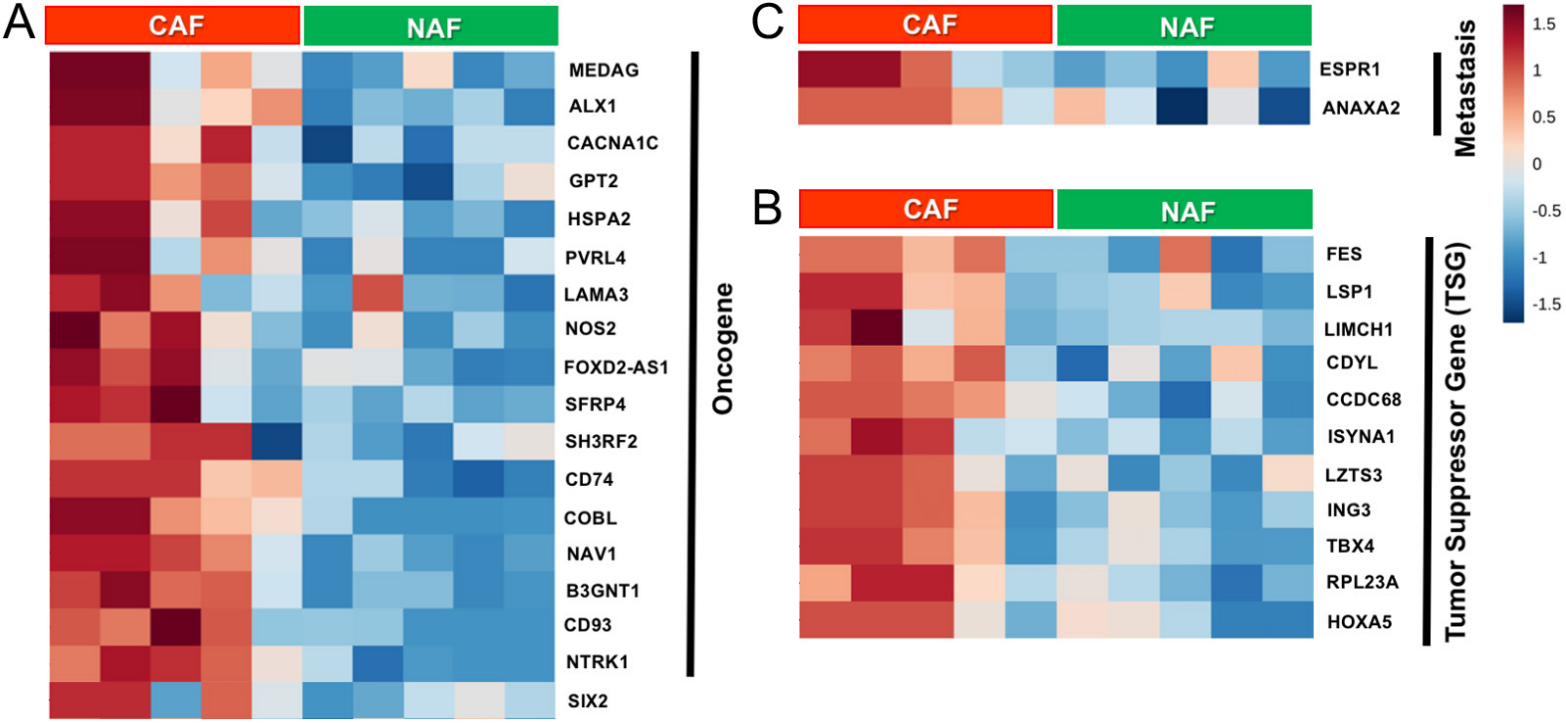
Heatmap summarizing DNA methylation levels of CpG repeats (blue color indicates hypomethylation and brown represents hypermethylation). (A) Hierarchical clustering and heatmap were generated for logarithmically transformed RRBS data and a columnwise normalization using MetaboAnalyst 3.0. (B) Tumor suppressor and (C) oncogenes identified from top 200 methylated genes differentially expressed between NAF and CAF are indicated. Each column represents a fibroblast sample, and each row represents the methylation level of indicated gene (*n* = 5).

Recent advancement in ‘omics’ technologies have allowed for genome-wide profiling of genome-scale DNA methylation both at a single-nucleotide and at a single-cell resolution (Lo & Zhou 2018). These methylation techniques are primarily based on the concept that treatment of sodium bisulfite on DNA leads to the conversion of nonmethylated cytosines to uracil whilst maintaining 5-methylcytosine (5mC) unchanged (commonly called as protected region) (Clark *et al.* 1994). Bisulphite conversion is still considered to be the ’gold standard’ to detect DNA methylation patterns. In addition, alternative methylcytosine-specific enrichment technologies, such as methylated DNA immunoprecipitation (MeDIP) and methyl-CpG-binding technologies are region-based approaches in whole genomes, therefore, do not deliver the detail of DNA methylation patterns (Bock *et al.* 2010). Incorporation of next-generation sequencing methods with bisulfite conversion is the basis for reduced representation (RRBS) or whole genome (WGBS) data to identify genome-wide CpG coverage (Harris *et al.* 2010). We performed first application of RRBS technology in analyzing DNA methylation pattern in fibroblasts (Mishra *et al.* 2018). Comparing the DNA methylome analysis of prostatic NAF and CAF, we recognized genes that had reported roles in tumor progression, suppression, and metastasis (Table 1). There were 18 tumor-promoting, 11 suppressing, 2 metastasis regulatory gene promoters’ hypermethylated in the prostatic CAFs. Heat maps of the genes suggest critical novel biomarkers for prostate cancer (Fig. 1). The rational for focusing on known tumor regulators in the non-transformed fibroblastic cells is based on significant evidence that such genes in fibroblasts have distinct paracrine effects on associated epithelia. Indeed, the forced expression of two oncogenic events are required to transform embryonic fibroblasts (Land *et al.* 1983). However, the effects on adjacent epithelia only seem to require a single such hit. For example, the loss of tumor suppressors, such as *TGFBR2* or phosphatase and tensin homolog (*PTEN*) in prostate and breast fibroblasts, respectively, has been associated with breast and prostate cancer mouse models (Bhowmick *et al.* 2004, Cheng *et al.* 2005, Trimboli *et al.* 2009). In parallel, oncogene expression of cyclin D1 (*CCND1*) and *CMYC* in the CAF has been reported to promote tumorigenicity in PCa models (He *et al.* 2007, Valencia *et al.* 2014, Minciacchi *et al.* 2017). In fact, gastric cancer-associated stromal methylation signature was found to be a determinant of epithelial tumor stage (Jiang *et al.* 2008). Methylation-sensitive SNP array analysis (MSNP) was used to compare DNA methylation in NAF and CAF cells. Fewer genes were found to have promoter hypermethylation in CAFs compared to NAF (Jiang *et al.* 2008). Aberrant DNA methylation pattern in CAFs that affected TGF-β signaling was found to be prognostic for non-small-cell lung cancer patients (Vizoso *et al.* 2015). CAF in pancreatic ductal adenocarcinoma, associated with extensive connective tissue deposition, had a distinct methylation landscape that promote malignant growth and progression. Suppressor of cytokine signaling (*SOCS*) family gene, SOCS1 was identified as a prominent gene frequently methylated in pancreatic CAFs (Xiao *et al.* 2016). Conversely, the ADAM12 gene promoter was hypomethylated in pancreatic CAFs (Yu *et al.* 2012). Together, these data demonstrate stromal DNA methylation status can impact cancer progression.

**Table 1:** Differential promoter methylation of genes in prostatic CAF and NAF cells with roles as oncogene, tumor suppressor, and metastasis.

Name	Description	Biological effect	Reference
Oncogene			
MEDAG	Mesenteric estrogen-dependent adipogenesis	Observed in almost all cases of papillary thyroid carcinomas. High expression was correlated with metastasis and poor disease-free survival.	(Song *et al.* 2019)
ALX1	Aristaless-like homeobox1	Induces EMT and cell invasion in ovarian cancer cells by promoting Snail expression.	(Yuan *et al.* 2013)
CACNA1C	Calcium voltage-gated channel subunit alpha1 C	Expression was directly regulated by miR-363 whose high expression is associated with worse prognosis in diffuse large B-cell lymphoma (DLBCL).	(Zhang *et al.* 2019)
GPT2	Glutamic pyruvate transaminase GPT2	Promotes tumorigenesis and stemness of breast cancer cells by activating the Shh signaling	(Cao *et al.* 2017)
HSPA2	Heat shock-related 70-kDa protein 2	Overexpression is correlated with tumor angiogenesis and poor prognosis in pancreatic carcinoma.	(Zhai *et al.* 2017)
PVRL4	Poliovirus-receptor-like 4	Associated with breast cancer transformation and involved in cell-to-cell attachment with monoclonal antibodies	(Pavlova *et al.* 2013)
LAMA3	Laminin alpha 3	The analysis identified a splice variant known to be involved in tumor cell invasion and progression.	(Moller-Levet *et al.* 2009)
NOS2	Nitric oxide synthase	Its expression was associated with brain metastases in mouse models of orthotopic breast cancer xenografts.	(Heinecke *et al.* 2014)
FOXD2-AS1	FOXD2 adjacent opposite strand RNA1	Promoted the progression of colorectal cancer by regulating EMT and Notch signaling pathway.	(Yang *et al.* 2017)
SFRP4	Secreted frizzled-related protein 4 (SFRP4)	Elevated gene expression is associated with high grade disease and recurrent prostate cancer after surgery.	(Sandsmark *et al.* 2017)
SH3RF2	SH3-domain-containing RING finger protein	Regulates p21-activated kinase 4 (PAK4) protein stability. Ectopic expression limit apoptosis and enhances cell migration, colony formation and tumor growth.	(Kim *et al.* 2014)
CD74	Cluster of Differentiation 93	In several forms of cancer, CD74 is up-regulated and associated with enhanced proliferation and metastatic potential	(Schroder 2016)
COBL	cordon-bleu WH2 repeat protein)	It is involved in the cancer cell morphogenesis, implicated in the acquisition of the neuron-like cell shape observed in neuroendocrine prostate cancer.	(Lopes *et al.* 2016, Takayama *et al.* 2018)
NAV1	Neuron navigator 1	Expressed in brain astrocytoma, its expression was positively correlated with the degree of malignancy	(Xing *et al.* 2014)
B3GNT1	β-1,3-N-acetylglucosaminyltransferase 1	Wild-type but not mutant B3GNT1 in human prostate cancer cells led to increased levels of α-dystroglycan glycosylation, associated with extracellular matrix.	(Buysse *et al.* 2013)
CD93	Cluster of Differentiation 93	A key regulator of glioma angiogenesis, acting via cytoskeletal rearrangements required for cell-cell and cell-matrix adhesion.	(Langenkamp *et al.* 2015)
NTRK1	Neurotrophic receptor tyrosine kinase 1	Tumor samples from 3 of 91 patients with lung cancer (3.3%) without known oncogenic alterations assayed by next-generation sequencing or fluorescence in situ hybridization demonstrated evidence of NTRK1 gene fusions	(Vaishnavi *et al.* 2013)
SIX2	SIX homeobox 2	Transcription factor involved in organ development and breast cancer stem cells through the positive regulation of SOX2	(Wang *et al.* 2014, Oliphant et al. 2019)
Tumor suppressor			
FES	c-fes protein-tyrosine kinase	Expression downregulated in colon tumors. Restoration of expression suppressed their colon cancer growth in soft agar.	(Delfino *et al.* 2006)
LSP1	Lymphocyte‐specific protein 1	Inhibits the growth of hepatocellular carcinoma by suppressing ERK1/2 phosphorylation. Patients with high LSP1 expression had significantly better overall survival.	(Zhang *et al.* 2016)
LIMCH1	Lim and calponin-homology domains 1	Potentiates actin stress fiber assembly and stabilizes focal adhesions to negatively regulate cell spreading and migration	(Lin *et al.* 2017)
CDYL	Chromodomain on y-like	CDYL bridges REST and histone methyltransferases for gene repression and suppression of cellular transformation. Loss of heterozygosity associated with cervical cancer transformation.	(Mulligan *et al.* 2008)
CCDC68	Coiled-coil domain containing 68	Allows for centriol anchoring to microtubules in interphase cells. Directly associated with pancreatic cancer proliferation.	(Radulovich *et al.* 2015)
ISYNA1	Inositol 3-phosphate synthase (ISYNA1)	Ectopic ISYNA1 expression increased myo-inositol levels in the cells and suppressed tumor cell growth.	(Koguchi *et al.* 2016)
LZTS3	Leucine zipper tumor suppressor family member 3	In silico characterization of LZTS3 identified its potential tumor suppressor.	(Teufel *et al.* 2005)
ING3	Inhibitor of growth	Can activate p53 trans-activated promoters, including promoters of p21/waf1 and Bax. Overexpression can inhibit cell growth and induce apoptosis in head and neck cancers	(Gou *et al.* 2014)
TBX4	T-box transcription factor Tbx4	Reduced expression suggests a worse prognosis for pancreatic cancer patients.	(Zong *et al.* 2011)
RPL23A	Ribosomal protein L23A gene	A component of the 60S ribosomal subunit exhibits anti-cancer function on the Hep-2 cells.	(Sun *et al.* 2012)
HOXA5	Homeobox A5	Loss of expression occurs frequently in breast cancer and correlates with higher pathological grade and poorer disease outcome.	(Teo *et al.* 2016)
Metastasis			
ESRP1	Epithelial splicing regulatory protein 1	Drives a switch from mesenchymal to epithelial phenotype characterized by reduced cell migration of ovarian cancer	(Jeong *et al.* 2017)
ANXA2	Annexin A2	High-affinity binding for Ca and phospholipids like other annexin family members. Implicated in multiple cancer types to greater metastasis and poor prognosis.	(Christensen *et al.* 2018, Li *et al.* 2019)

**Figure 2:**
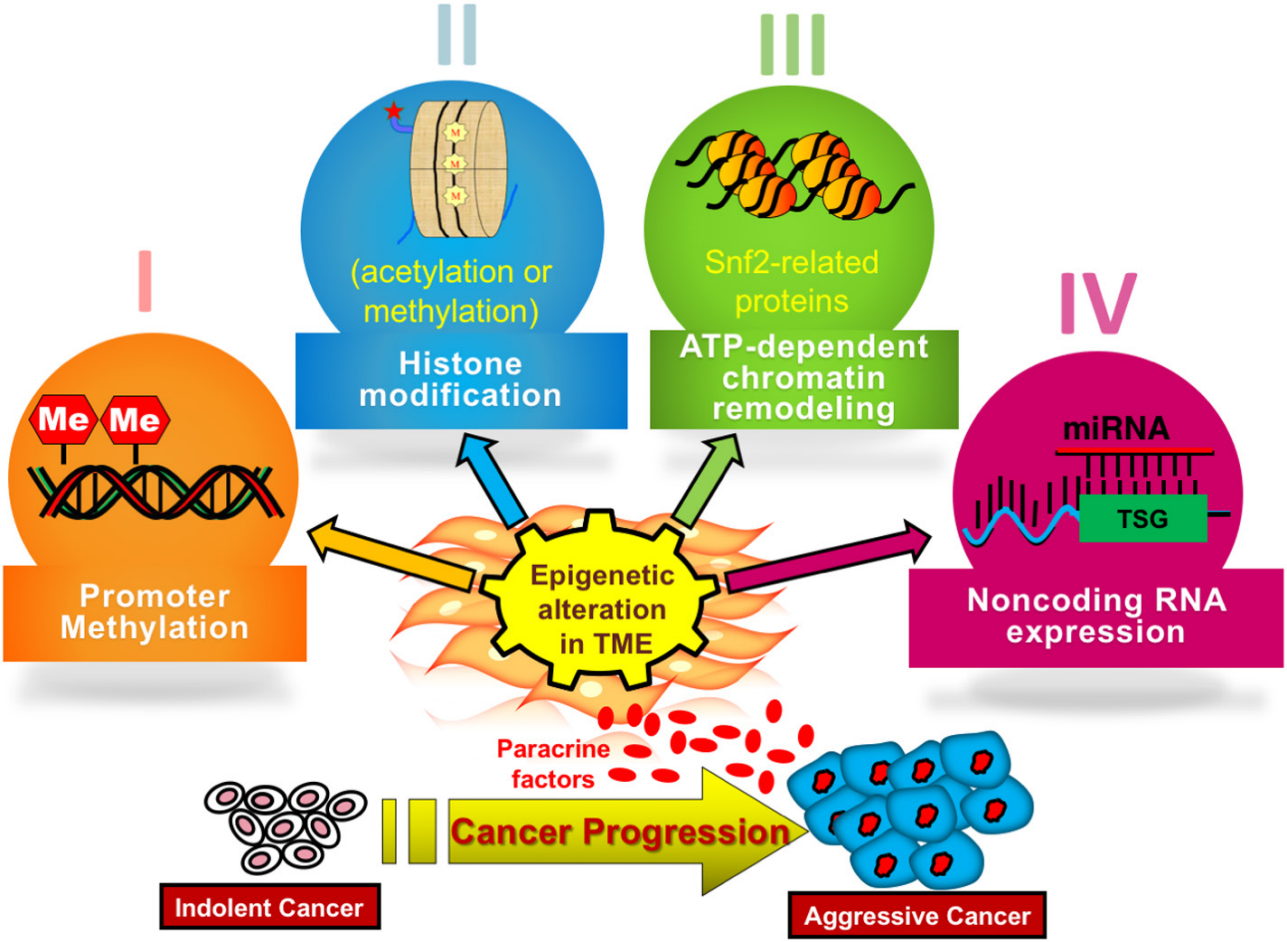
A general scheme of epigenetic changes in fibroblasts include four basic mechanisms: (I) promoter DNA modifications, (II) histone modifications, (III) chromatin remodeling with polycomb proteins, and (IV) aberrant expression of miRNA. These well-known epigenetic modifications taking place in the tumor microenvironment can lead to transcriptomic changes, that in-turn can be suppressive of promoting of tumor expansion in a paracrine manner.

In a noteworthy study, Albrengues *et al.* demonstrated that an epigenetic switch involving the leukemia-inducible factor (LIF), a proinflammatory cytokine of IL-6 class secreted by cancer cells, reprograms human head and neck CAF into a state that supported cancer cell invasion via extracellular matrix (ECM) remodeling (Albrengues *et al.* 2015). They further showed that DNMT3B methylated CpG sites of the SHP-1 phosphatase promoter to downregulate SHP-1 expression, resulting in constitutive phosphorylation of JAK1. Thereafter JAK1/STAT3 signaling was sustained by maintenance methylation enzyme, DNMT1. This study provided a unique link of histone modification and DNA methylation in fibroblasts. The authors observed that DNMT inhibitor, 5-AzaDC, restored the expression of SHP-1, thereby decreasing JAK1/STAT3 activation, and tumor-inductive properties of the fibroblasts. All together, these studies demonstrated crucial role of DNA methylation activity of the tumor microenvironment provided sustained head and neck cancer proinvasive activity. Histone methylation is also crucial for fibroblast activation. Accordingly, Tyan *et al.* reported that the loss of EZH2 (enhancer of zeste homolog 2) caused promoter-associated histone H3K27 methylation at the ADAMTS1 gene (ADAM metallopeptidase with thrombospondin type 1 motif), accounting for its enhanced expression (Tyan *et al.* 2012). These studies supported the role of epigenetic modification in breast stromal fibroblasts in conferring a tumor-inductive phenotype. Apart from histone modification, non-histone chromatin remodeling gene, Hmga2 (High-mobility group AT-hook 2) has been identified as an epigenetic regulator in prostatic fibroblasts. Stromal-specific overexpression of Hmga2 in mouse fibroblasts was sufficient for the induction of multifocal prostatic intraepithelial neoplasia in adjacent prostatic epithelia (Zong *et al.* 2012). More research is needed to understand the underpinning mechanisms for the emergence of the stable CAF phenotype. Figure 2 illustrates general epigenetic changes involved in fibroblast which alter cancer epithelial communications and proliferations.

## Epigenetic silencing of RasGAPs: alternative route to Ras signaling activation in cancer

**Figure 3:**
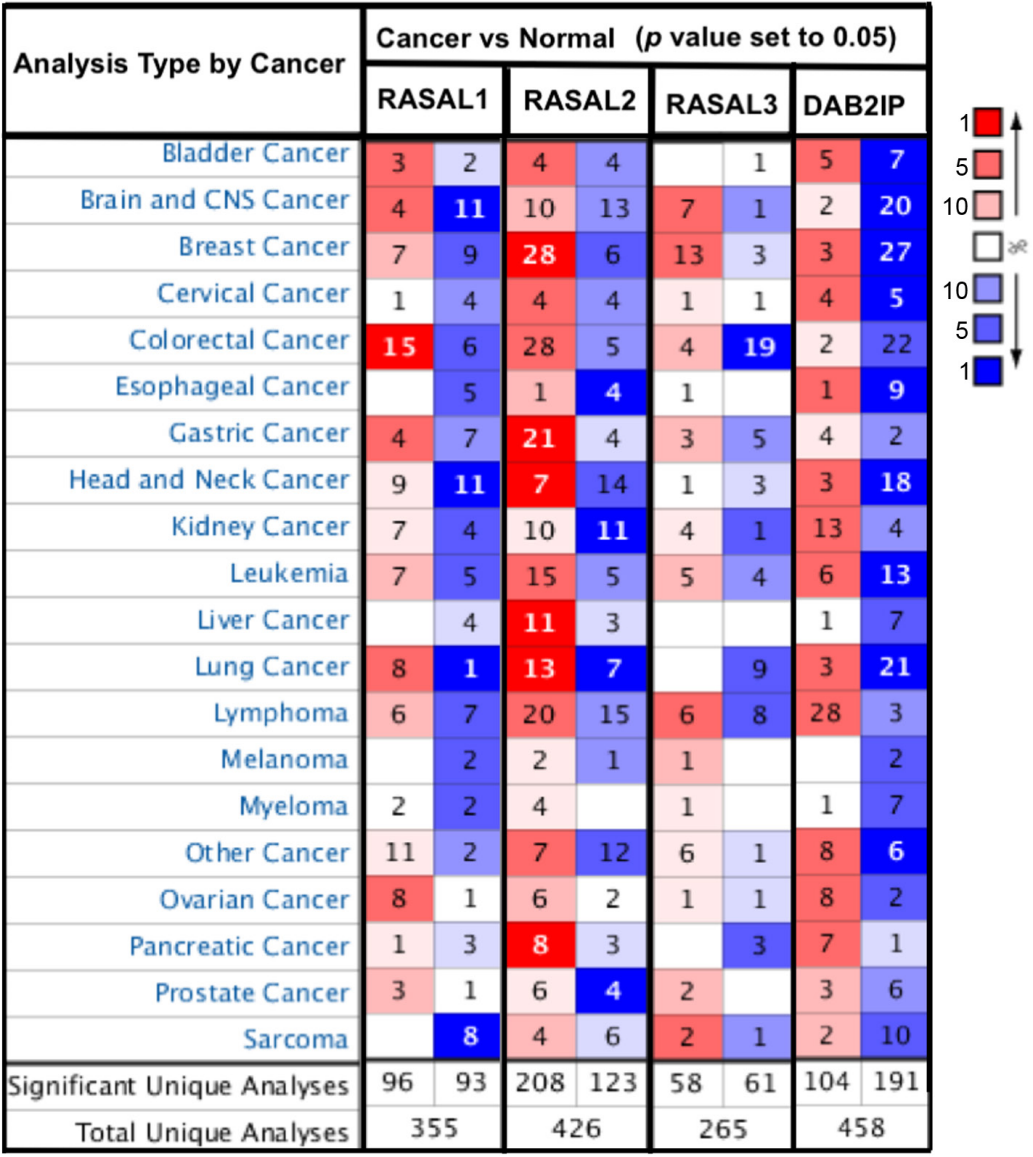
The expression levels of human RASAL1, RASAL2, RASAL3 and DAB2IP are profiled across multiple cancer types, compared to normal tissue by Oncomine. The gene expression level differences between cancer and normal tissue are illustrated. The number of datasets in which statistically significant mRNA overexpression or under-expression was observed is indicated in red or blue boxes, respectively. The color intensity corresponds to the gene rank and magnitude of expression differences with a statistically significant threshold.

Altered Ras signaling has achieved notoriety in contributing to tumorigenesis (Fernandez-Medarde & Santos 2011). More than 30% of all human neoplasms harbor an oncogenic form of Ras proteins, made up of a small family of three closely related proteins (K-Ras, H-Ras, or N-Ras) (Adjei 2001, Canevari *et al.* 2002). As GTPases, Ras proteins oscillate between an active GTP-bound and guanosine diphosphate (GDP)-bound inactive state. The RasGAP family of proteins inactivate Ras signaling by binding Ras and catalyzing Ras-GTP hydrolysis to Ras-GDP (King *et al.* 2013, Simanshu *et al.* 2017, Scheffzek & Shivalingaiah 2019). The silencing of the RasGAP genes by promoter methylation results in the activation of RAS signaling and promote primary tumor development (Fernandez-Medarde & Santos 2011, Simanshu *et al.* 2017). In addition, the inactivation of the RasGAP, RASAL1, in fibroblasts can contribute to renal and cardiac fibrosis (Bechtel *et al.* 2010, Xu *et al.* 2015). There are 14 RasGAP genes identified in the human genome (Bernards 2003). We performed Oncomine analysis to investigate the differences in the mRNA levels of different RasGAPs genes, between tumor and normal tissues in multiple cancer types (Fig. 3). The epigenetic regulation of RasGAP proteins that contribute to activation of Ras signaling and its implication in tumorigenesis is further discussed below.

DAB2IP is one of most well-studied RasGAPs in cancers, also known as AIP1 (ASK1-interacting protein). Several studies reported *DAB2IP* gene regulation through aberrant methylation in prostate, breast, lung, liver and gastrointestinal cancers (Chen *et al.* 2003, Dote *et al.* 2004, 2005, Yano *et al.* 2005). A DNA methylation-based study conducted in renal cell carcinoma identified DAB2IP promoter methylation as a practical prognostic biomarker. The CpG methylation biomarker is located upstream of the transcription start site of *DAB2IP* (DAB2IP CpG1). Pyrosequencing quantitative methylation assay of over 550 patient paraffin renal cancer tissue sections was used to establish a correlation between DAB2IP CpG1 methylation and overall survival (Wang *et al.* 2016). Similarly, *DAB2IP* promoter methylation and expression downregulation were identified to be associated with breast cancer lymph node metastasis (Dote *et al.* 2004). The restoration of DAB2IP expression by 5-acetazolamide-2-cytosine deoxyriboside (5azaDC, DNA demethylating agent) supported the epigenetic regulation of breast cancer progression (Dote *et al.* 2004). Methylation of *DAB2IP* exon 3 was associated with histone H3 di- and trimethyl H3-Lys27 (H3K27me2 and H3K27me3), a site known to be modified by EZH2 and recruitment of polycomb repressive complex 2 and histone deacetylases (Chen *et al.* 2003, Smits *et al.* 2012). The established tumor-suppressive role of DAB2IP has been extended to its role in angiogenesis inhibition and chemo/radiation sensitization, to reveal some Ras-independent effects of this RasGAP.

RASAL1 has been identified as a tumor suppressor, frequently silenced by promoter hypermethylation in numerous cancer types. For example, screening of 13 RasGAPs in 12 human thyroid cancer cell lines revealed epigenetic silencing of *RASAL1* (Liu *et al.* 2013). Notably, treating these cell lines with 5azaDC restored *RASAL1* expression. In another example, promoter hypermethylation of RASAL1 was found in colorectal cancers, interestingly frequently also associated with K-Ras mutational activation (Ohta *et al.* 2009). Ectopic expression of RASAL1 or using a DNA methylation inhibitor was found to reduce Ras signaling and colon cancer progression (Liu *et al.* 2005, Ohta *et al.* 2009). Likewise, RASAL1 promoter DNA hypermethylation in gastric cancer tumor tissues were greater than that in paired adjacent non-tumor tissues (Chen *et al.* 2013). Apart from DNA methyltransferases, there are also histone-modifying enzymes, which can play a role in the regulation of RASAL1. Brigette *et al.* revealed that treatment with histone deacetylase inhibitor (HDACi), belinostat (PXD101), led to a modest restoration of RASAL1 expression in HepG2 and Hep3b cell lines (Ma *et al.* 2010). For these diverse cancer types with RASAL1 epigenetic silencing, often associated with Ras-driven carcinogenesis, the added loss of the suppressor potentially super-activates the Ras signaling axis. In the same models, however, the restoration of RASAL1 expression was found to negate some of the effects of the endogenous Ras-activating mutations or amplification. Thus, epigenetically regulated RasGAP activity can be considered to be dominant over such genomic alterations of the *Ras* gene.

Epigenetic silencing of RASAL2 has demonstrated that it can function as a tumor and metastasis suppressor, in breast cancer, hepatocellular carcinoma, colorectal cancer (Jia *et al.* 2017), nasopharyngeal carcinoma, lung cancer, and ovarian cancer (McLaughlin *et al.* 2013, Feng *et al.* 2014, Huang *et al.* 2014, Li & Li 2014, Stefanska *et al.* 2014, Wang *et al.* 2015, Yan *et al.* 2016, Olsen *et al.* 2017). Notably, promoter hypermethylation of *RASAL2* and *DAB2IP* was identified in aggressive luminal B breast cancer (Olsen *et al.* 2017). Performing gain-of-function and loss-of-function studies, Hui *et al.* demonstrated that formation of new blood vessels was suppressed by RASAL2 via VEGFA downregulation in renal cell carcinoma metastasis (Hui *et al.* 2018). Further, the epigenetic silencing of RASAL2 was negatively correlated with the overall survival of renal cell carcinoma patients (Hui *et al.* 2018).

Unlike the other two RASAL family members, RASAL3 has not been considered a tumor suppressor in the traditional sense, in terms of being silenced in tumor cells. The role of RASAL3 in immune cells have been recognized. Initially, the epigenetic silencing of Rasal3 was observed in canine B-cell lymphoma, identified by DNA methylome by genome-wide CpG microarray (Stefanska *et al.* 2014). Subsequently, a mouse model systemically knocking out Rasal3 resulted in reduced number of natural killer (NK) cells and diminished expression of interleukin-4 and interferon-γ by the NK cells (Saito *et al.* 2015). The knockout of *Rasal3* also results in reduced number of naïve T cells, demonstrating the role of RASAL3 in supporting cell survival (Muro *et al.* 2018). In light of the recognized importance of tumor immunity, there may be further justification for the restoration of RASAL3 expression, potentially through the use of HDAC inhibitors or DNA-demethylating agents. In fact, the current use and observed efficacy of such therapeutics may in part be due to their impact on RASAL3 on non-tumor cells. We reported RASAL3 promoter methylation, through RRBS sequencing analysis, is a crucial step in activating Ras signaling in prostatic CAF (Mishra *et al.* 2018). As described earlier, oncogene signaling in CAF can potentiate the expansion of adjacent cancer epithelia. Accordingly, we found that active Ras signaling in the CAF caused PCa epithelial proliferation and acquisition of a neuroendocrine phenotype. Interestingly, we further revealed that RASAL3 epigenetic silencing and Ras signaling activation in CAF was heightened by the androgen receptor antagonism, a mainstay in PCa therapy. It is important to note that PCa is not recognized as a Ras-driven cancer. Hormone signaling regulates oncogenic signaling mechanisms that stimulate the activation of fibroblasts, can cause therapeutic resistance of the adjacent epithelia in a paracrine manner.

## Enhanced macropinocytosis provide metabolic flexibility for tumor cells

In Ras-driven cancers like pancreatic and glioblastoma, a process of uptake of albumin and other macromolecules from its surroundings, termed macropinocytosis occurs (Commisso *et al.* 2013, Muller-Greven *et al.* 2017). Subsequent albumin translocation to lysosomes generates amino acids. And as albumin is rich in glutamine, an outcome of macropinocytosis is glutamine efflux. For pancreatic cancer, the further metabolism of glutamine serves as a means of fueling cancer progression. However, we found that prostatic CAFs do not seem to metabolize the glutamine further (Mishra *et al.* 2018). Rather, glutamine gets secreted for its uptake by adjacent cancer epithelia, where it is metabolized to glutamate and enters the TCA cycle via α-ketoglutarate. A key hurdle for cancer cells is to fulfill rising energy demand for the growing biomass in often nutrient-depleted conditions (DeBerardinis & Chandel 2016). In order to meet energy/biosynthetic demand, tumors have evolved tremendous capacity to reprogram pathways triggering nutrient acquisition. Metabolic reprogramming is recognized as one of the hallmarks of cancer (Hanahan & Weinberg 2011) and explored as therapeutic targets (Altman *et al.* 2016, DeBerardinis & Chandel 2016, Cluntun *et al.* 2017). We demonstrated that the uptake of glutamine by amino acid transporter (SLC1A5), as well the metabolism of glutamine to glutamate by glutaminase (GLS) was upregulated in the cancer epithelia in response to elevated concentrations of glutamine in the media (Mishra *et al.* 2018). Macropinocytosis is one of the important strategies that cancer cells use as an alternative nutrient acquisition pathway (Commisso *et al.* 2013, Zwartkruis & Burgering 2013, Nakase *et al.* 2015, Wang *et al.* 2018). While the first microscopic observations of macropinocytosis in malignant cells was discovered in 1930s, its mechanistic understanding occurred in the last few years. The uptake of macromolecules through a specialized process of plasma membrane ruffling for the formation of endocytic macropinosomes that fuse into lysosomes is now an established process for anabolic metabolism for cancer cells (Recouvreux & Commisso 2017, Wang *et al.* 2018). Apart from oncogenic Ras activation, phosphatidylinositol 3-kinase (PI3-kinase) and phosphatase and tensin homolog (PTEN) mutations found in cancer (Chalhoub & Baker 2009) may also potentiate macropinocytosis as an adaptation to limiting nutrient availability (DeBerardinis & Chandel 2016, Cluntun *et al.* 2017, Recouvreux & Commisso 2017). However, in the case of PCa, the stromal co-evolution with the cancer epithelia involve epigenetic imprinting associated with *RASAL3* silencing. This particular stromal reprogramming supports cancer progression via the induction of fibroblastic activation and secretion of glutamine.

The role of glutamine as a conditionally essential amino acid for cancer cells is well documented as a critical metabolite for nucleotide biosynthesis and anaplerosis. In addition, we found that incubation of PCa cells with glutamine resulted in the expression of neuroendocrine markers. We demonstrated that the uptake and metabolism of glutamine by SLC1A5 and GLS, respectively, was critical to the differentiation of prostate adenocarcinoma to the neuroendocrine phenotype. Neuroendocrine prostate cancer (NEPC) cells loose granular structure and tend to have a small cell-like morphology characterized by the varying levels of expression of neuronal markers, including chromogranin A (CGA), synaptophysin (SYP), neurospecific enolase (NSE), and more recently T-Box brachyury (Blaschko *et al.* 1967, Schmechel *et al.* 1978, Wiedenmann *et al.* 1986, Pinto *et al.* 2016). *De novo* NEPC is one of the rarest form (<1%) of the disease (Gupta & Gupta 2017). However, in response to AR signaling inhibition and/or androgen deprivation therapy, transdifferentiation to NEPC can support rapid disease progression with universally poor outcome, with an overall 5-year survival rate of 12.6% (Beltran *et al.* 2014, Yadav *et al.* 2016). Importantly, the transdifferentiated NEPC does not necessarily exhibit all the characteristics of *de novo* NEPC (Beltran *et al.* 2012, 2014). For example, transdifferentiated NEPC often maintains responsiveness to androgens despite its resistance to AR signaling inhibitors. While its incidence in primary prostate cancers is exceedingly low, in metastatic castrate-resistant prostate cancers (CRPCs), its percentage goes up to 25–30% (Gupta & Gupta 2017). Paracrine glutamine signaling is a mechanism by which AR signaling inhibitors potentiate this phenotype. We validated this finding in PCa patients that were on androgen receptor signaling inhibitors and found that those patients that developed therapeutic resistance had significantly higher blood glutamine levels compared to those who remained sensitive to hormone therapy (Mishra *et al.* 2018).

**Figure 4:**
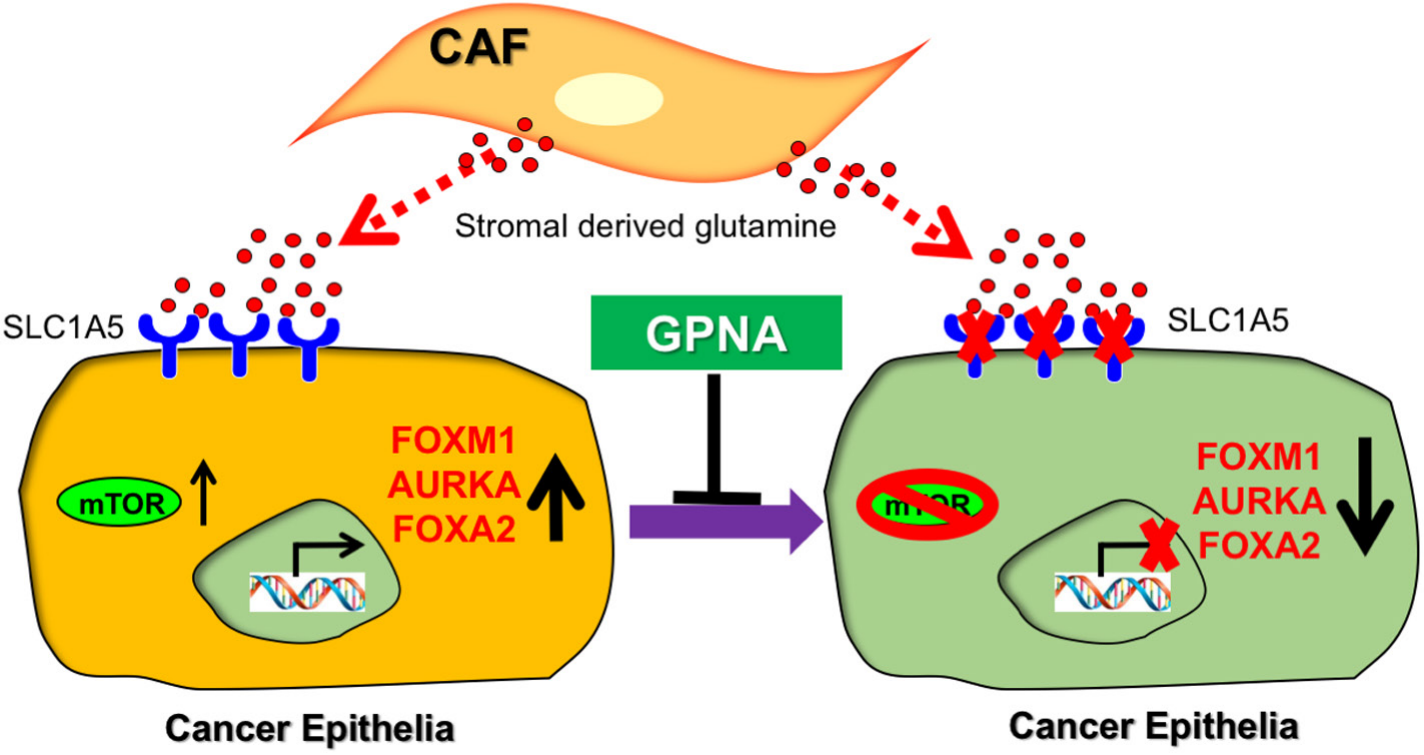
Proposed model of stromal induced-neuroendocrine prostate cancer (NEPC). Carcinoma-associated fibroblasts (CAFs)-derived glutamine that can be taken-up by glutamine transporter, SLC1A5, and result in elevated mTOR signaling. Typical disease markers including chromogranin A (CHGA), FOXM1 and FOXA2 are shown upregulated after glutamine uptake in response to mTOR signaling. Inhibition of glutamine uptake by using SLC1A5 inhibitor, GPNA, limit the expression of NEPC markers. The studies suggest the importance of glutamine in NEPC transdifferentiation of prostate adenocarcinoma (Mishra *et al.* 2018).

## Therapeutic interventions in response to stromal co-evolution

There is a need for better understanding of NEPC with the approval of more effective inhibitors of AR signaling (i.e. enzalutamide, apalutamide, darolutamide) for advanced PCa. Genomic characterization of transdifferentiated NEPC phenotypic tumors revealed recurrent amplifications of *MYCN* and *AURKA* as well as lesions of *RB1* and *TP53* (Beltran *et al.* 2011, Tan *et al.* 2014). For example, *MYCN* mutations are found in 40% of NEPCs, but only observed in 5% of all other PCa (Beltran *et al.* 2011). However, expression of other recognized NEPC markers, *CHGA*, *SYP*, *NCAM1*, and *ENO2*, was heterogeneous. The role of biomarkers not only serve to characterize the tumor type, but may provide a clue as to an effective intervention. Aurora kinase (AURK) was a specifically targeted kinase for cancers driven by *MYCN*, such as NEPC, neuroblastoma, and hepatocellular carcinoma with significant efficacy in mouse models (Otto *et al.* 2009, Dauch *et al.* 2016, Lee *et al.* 2016). Since AURK was found to bind and stabilization of MYCN (Otto *et al.* 2009), its inhibition resulted in MYCN degradation and reduction in tumor volume in a model of NEPC (Lee *et al.* 2016). A subsequent phase II clinical trial for NEPC patients with a AURK inhibitor, alisertib, unfortunately did not meet its primary endpoint, but the subset of patients that exhibited elevated MYCN and AURK were found to gain significant clinical benefit (Beltran *et al.* 2019).

*MYC* amplification can contribute to the regulation of glutamine metabolism in prostate cancer. Cancers with *MYC* amplification exhibit elevated expression of amino acid transporters SLC1A5 and SLC38A5, as well as glutamine-metabolizing enzyme, GLS. Glutamine addiction of cancer cells can be exploited through the inhibition of amino acid transporters or inhibitors of glutamine metabolism. However, non-cancer cells are generally non-vulnerable to such glutamine deprivation (Chen & Cui 2015, Altman *et al.* 2016, Still & Yuneva 2017). Understanding of compensatory pathways of glutamine metabolism may improve the efficacy of cancer treatments. A sensor for abundant ATP includes the inhibition of AMP kinase, a known blocker of mTOR signaling. Thus, glutamine metabolism causes inhibition of the inhibitor of mTOR, resulting in mTOR activation and its downstream transcription factor FOXM1 in potentiating the expression of a number of NEPC-associated genes (Mishra *et al.* 2018). FOXM1 is a known master regulator of cancer metastasis, the expression of multiple stem cell genes, as well as *MYCN* and *AURK* (Raychaudhuri & Park 2011). Blocking GLS with BPTES (bis-2-(5-phenylacetamido-1,3,4-thiadiazol-2-yl)ethyl sulfide) inhibited NEPC transdifferentiation (Mishra *et al.* 2018) (Fig. 4). CB-839 is a glutaminase inhibitor that is shown to be safe in phase I clinical trials and considerably more potent than BPTES. Blocking glutamine uptake by using GPNA (l-γ-glutamyl-p-nitroanilide), a SLC1A5 inhibitor was effective in reducing tumor growth in the context of a commonly administered androgen receptor signaling inhibitor, enzalutamide (Mishra *et al.* 2018). As metastatic castrate-resistant prostate tumors have elevated available glutamine in circulation and its uptake can potentiate resistance to current AR signaling inhibition, a richer understanding of this pathway would contribute to better PCa treatment strategies.

## Declaration of interest

The authors declare that there is no conflict of interest that could be perceived as prejudicing the impartiality of this review.

## Funding

This work was supported by the Department of Defense (W81XWH-19-1-0388) and Veterans Affairs (I01BX001040) to N A B, as well as a Manipal University Jaipur, Enhanced Seed grant (EF/2018-19/QE04-11) to R M.

## References

[bib1] Adjei AA 2001 Blocking oncogenic Ras signaling for cancer therapy. Journal of the National Cancer Institute 1062–1074. (10.1093/jnci/93.14.1062) 11459867

[bib2] Albrengues J Bertero T Grasset E Bonan S Maiel M Bourget I Philippe C Herraiz Serrano C Benamar S Croce O, 2015 Epigenetic switch drives the conversion of fibroblasts into proinvasive cancer-associated fibroblasts. Nature Communications 10204 (10.1038/ncomms10204) PMC4682161 26667266

[bib3] Allocati N Masulli M Di Ilio C Federici L 2018 Glutathione transferases: substrates, inhibitors and pro-drugs in cancer and neurodegenerative diseases. Oncogenesis 8 (10.1038/s41389-017-0025-3) 29362397 PMC5833873

[bib4] Altman BJ Stine ZE Dang CV 2016 From Krebs to clinic: glutamine metabolism to cancer therapy. Nature Reviews: Cancer 749 (10.1038/nrc.2016.114) 28704361

[bib5] Banerjee J Mishra R Li X Jackson RS 2nd Sharma A Bhowmick NA 2014 A reciprocal role of prostate cancer on stromal DNA damage. Oncogene 4924–4931. (10.1038/onc.2013.431) 24141771 PMC4121379

[CIT131] Bechtel W McGoohan S Zeisberg EM Muller GA Kalbacher H Salant DJ Muller CA Kalluri R Zeisberg M 2010 Methylation determines fibroblast activation and fibrogenesis in the kidney. Nature Medicine 16 544–550. 10.1038/nm.2135 PMC3106179 20418885

[bib7] Beltran H Rickman DS Park K Chae SS Sboner A MacDonald TY Wang Y Sheikh KL Terry S Tagawa ST, 2011 Molecular characterization of neuroendocrine prostate cancer and identification of new drug targets. Cancer Discovery 487–495. (10.1158/2159-8290.CD-11-0130) 22389870 PMC3290518

[bib8] Beltran H Tagawa ST Park K MacDonald T Milowsky MI Mosquera JM Rubin MA Nanus DM 2012 Challenges in recognizing treatment-related neuroendocrine prostate cancer. Journal of Clinical Oncology e386–e389. (10.1200/JCO.2011.41.5166) 23169519

[bib9] Beltran H Tomlins S Aparicio A Arora V Rickman D Ayala G Huang J True L Gleave ME Soule H, 2014 Aggressive variants of castration-resistant prostate cancer. Clinical Cancer Research 2846–2850. (10.1158/1078-0432.CCR-13-3309) 24727321 PMC4040316

[bib6] Beltran H Oromendia C Danila DC Montgomery B Hoimes C Szmulewitz RZ Vaishampayan U Armstrong AJ Stein M Pinski J, 2019 A phase II trial of the aurora kinase a inhibitor alisertib for patients with castration-resistant and neuroendocrine prostate cancer: efficacy and biomarkers. Clinical Cancer Research 43–51. (10.1158/1078-0432.CCR-18-1912) 30232224 PMC6320304

[CIT132] Bernards A 2003 GAPs galore! A survey of putative Ras superfamily GTPase activating proteins in man and Drosophila. Biochimica et Biophysica Acta 1603 47–82. 12618308 10.1016/s0304-419x(02)00082-3

[bib10] Bhowmick NA Chytil A Plieth D Gorska AE Dumont N Shappell S Washington MK Neilson EG Moses HL 2004 TGF-beta signaling in fibroblasts modulates the oncogenic potential of adjacent epithelia. Science 848–851. (10.1126/science.1090922) 14764882

[bib11] Bianchi-Frias D Basom R Delrow JJ Coleman IM Dakhova O Qu X Fang M Franco OE Ericson NG Bielas JH, 2016 Cells comprising the prostate cancer microenvironment lack recurrent clonal somatic genomic aberrations. Molecular Cancer Research 374–384. (10.1158/1541-7786.MCR-15-0330) 26753621 PMC5582956

[bib12] Blaschko H Comline RS Schneider FH Silver M Smith AD 1967 Secretion of a chromaffin granule protein, chromogranin, from the adrenal gland after splanchnic stimulation. Nature 58–59. (10.1038/215058a0) 6053402

[bib13] Bock C Tomazou EM Brinkman AB Muller F Simmer F Gu H Jager N Gnirke A Stunnenberg HG Meissner A 2010 Quantitative comparison of genome-wide DNA methylation mapping technologies. Nature Biotechnology 1106–1114. (10.1038/nbt.1681) PMC3066564 20852634

[CIT133] Buysse K Riemersma M Powell G van Reeuwijk J Chitayat D Roscioli T Kamsteeg EJ van den Elzen C van Beusekom E Blaser S, *et al* 2013 Missense mutations in beta-1,3-N-acetylglucosaminyltransferase 1 (B3GNT1) cause Walker-Warburg syndrome. Human Molecular Genetics 22 1746–1754. 23359570 10.1093/hmg/ddt021 PMC3613162

[bib14] Canevari S Biocca S Figini M 2002 Re: blocking oncogenic Ras signaling for cancer therapy. Journal of the National Cancer Institute 1031–1032; author reply 1032. (10.1093/jnci/94.13.1031) 12096090

[CIT134] Cao Y Lin SH Wang Y Chin YE Kang L Mi J 2017 Glutamic pyruvate transaminase GPT2 promotes tumorigenesis of breast cancer cells by activating sonic hedgehog signaling. Theranostics 7 3021–3033. 28839461 10.7150/thno.18992 PMC5566103

[bib15] Chaffer CL Weinberg RA 2011 A perspective on cancer cell metastasis. Science 1559–1564. (10.1126/science.1203543) 21436443

[bib16] Chalhoub N Baker SJ 2009 PTEN and the PI3-kinase pathway in cancer. Annual Review of Pathology 127–150. (10.1146/annurev.pathol.4.110807.092311) PMC2710138 18767981

[bib19] Chen L Cui H 2015 Targeting glutamine induces apoptosis: a cancer therapy approach. International Journal of Molecular Sciences 22830–22855. (10.3390/ijms160922830) 26402672 PMC4613338

[bib18] Chen H Toyooka S Gazdar AF Hsieh JT 2003 Epigenetic regulation of a novel tumor suppressor gene (hDAB2IP) in prostate cancer cell lines. Journal of Biological Chemistry 3121–3130. (10.1074/jbc.M208230200) 12446720

[bib17] Chen H Pan Y Cheng ZY Wang Z Liu Y Zhao ZJ Fan H 2013 Hypermethylation and clinicopathological significance of RASAL1 gene in gastric cancer. Asian Pacific Journal of Cancer Prevention 6261–6265. (10.7314/apjcp.2013.14.11.6261) 24377515

[bib20] Cheng N Bhowmick NA Chytil A Gorksa AE Brown KA Muraoka R Arteaga CL Neilson EG Hayward SW Moses HL 2005 Loss of TGF-beta type II receptor in fibroblasts promotes mammary carcinoma growth and invasion through upregulation of TGF-alpha-, MSP- and HGF-mediated signaling networks. Oncogene 5053–5068. (10.1038/sj.onc.1208685) 15856015 PMC3074577

[CIT135] Christensen MV Hogdall CK Jochumsen KM Hogdall EVS 2018 Annexin A2 and cancer: a systematic review. International Journal of Oncology 52 5–18. 29115416 10.3892/ijo.2017.4197

[bib21] Clark SJ Harrison J Paul CL Frommer M 1994 High sensitivity mapping of methylated cytosines. Nucleic Acids Research 2990–2997. (10.1093/nar/22.15.2990) 8065911 PMC310266

[bib22] Cluntun AA Lukey MJ Cerione RA Locasale JW 2017 Glutamine metabolism in cancer: understanding the heterogeneity. Trends in Cancer 169–180. (10.1016/j.trecan.2017.01.005) 28393116 PMC5383348

[bib23] Commisso C Davidson SM Soydaner-Azeloglu RG Parker SJ Kamphorst JJ Hackett S Grabocka E Nofal M Drebin JA Thompson CB, 2013 Macropinocytosis of protein is an amino acid supply route in Ras-transformed cells. Nature 633–637. (10.1038/nature12138) PMC3810415 23665962

[bib24] Dauch D Rudalska R Cossa G Nault JC Kang TW Wuestefeld T Hohmeyer A Imbeaud S Yevsa T Hoenicke L, 2016 A MYC-aurora kinase A protein complex represents an actionable drug target in p53-altered liver cancer. Nature Medicine 744–753. (10.1038/nm.4107) 27213815

[bib25] DeBerardinis RJ Chandel NS 2016 Fundamentals of cancer metabolism. Science Advances e1600200 (10.1126/sciadv.1600200) 27386546 PMC4928883

[CIT136] Delfino FJ Stevenson H Smithgall TE 2006 A growth-suppressive function for the c-fes protein-tyrosine kinase in colorectal cancer. Journal of Biological Chemistry 281 8829–8835. 16455651 10.1074/jbc.M507331200

[bib26] Dey P 2011 Epigenetic changes in tumor microenvironment. Indian Journal of Cancer 507–512. (10.4103/0019-509X.92246) 22293269

[bib28] Dote H Toyooka S Tsukuda K Yano M Ouchida M Doihara H Suzuki M Chen H Hsieh JT Gazdar AF, 2004 Aberrant promoter methylation in human DAB2 interactive protein (hDAB2IP) gene in breast cancer. Clinical Cancer Research 2082–2089. (10.1158/1078-0432.CCR-03-0236) 15041729

[bib27] Dote H Toyooka S Tsukuda K Yano M Ota T Murakami M Naito M Toyota M Gazdar AF Shimizu N 2005 Aberrant promoter methylation in human DAB2 interactive protein (hDAB2IP) gene in gastrointestinal tumour. British Journal of Cancer 1117–1125. (10.1038/sj.bjc.6602458) PMC2361938 15770214

[bib29] Dumont N Wilson MB Crawford YG Reynolds PA Sigaroudinia M Tlsty TD 2008 Sustained induction of epithelial to mesenchymal transition activates DNA methylation of genes silenced in basal-like breast cancers. PNAS 14867–14872. (10.1073/pnas.0807146105) 18806226 PMC2567459

[bib30] Eads CA Nickel AE Laird PW 2002 Complete genetic suppression of polyp formation and reduction of CpG-island hypermethylation in Apc(Min/+) Dnmt1-hypomorphic mice. Cancer Research 1296–1299. 11888894

[bib31] Egger G Jeong S Escobar SG Cortez CC Li TW Saito Y Yoo CB Jones PA Liang G 2006 Identification of DNMT1 (DNA methyltransferase 1) hypomorphs in somatic knockouts suggests an essential role for DNMT1 in cell survival. PNAS 14080–14085. (10.1073/pnas.0604602103) 16963560 PMC1599915

[bib32] El-Osta A Wolffe AP 2000 DNA methylation and histone deacetylation in the control of gene expression: basic biochemistry to human development and disease. Gene Expression 63–75. (10.3727/000000001783992731) 11097425 PMC5964960

[bib33] Feng M Bao Y Li Z Li J Gong M Lam S Wang J Marzese DM Donovan N Tan EY, 2014 RASAL2 activates RAC1 to promote triple-negative breast cancer progression. Journal of Clinical Investigation 5291–5304. (10.1172/JCI76711) 25384218 PMC4348963

[bib34] Fernandez-Medarde A Santos E 2011 Ras in cancer and developmental diseases. Genes and Cancer 344–358. (10.1177/1947601911411084) 21779504 PMC3128640

[bib35] Fiori ME Di Franco S Villanova L Bianca P Stassi G De Maria R 2019 Cancer-associated fibroblasts as abettors of tumor progression at the crossroads of EMT and therapy resistance. Molecular Cancer 70 (10.1186/s12943-019-0994-2) 30927908 PMC6441236

[bib36] Gascard P Tlsty TD 2016 Carcinoma-associated fibroblasts: orchestrating the composition of malignancy. Genes and Development 1002–1019. (10.1101/gad.279737.116) 27151975 PMC4863733

[bib37] Goll MG Kirpekar F Maggert KA Yoder JA Hsieh CL Zhang X Golic KG Jacobsen SE Bestor TH 2006 Methylation of tRNAAsp by the DNA methyltransferase homolog Dnmt2. Science 395–398. (10.1126/science.1120976) 16424344

[bib38] Gordetsky J Epstein J 2016 Grading of prostatic adenocarcinoma: current state and prognostic implications. Diagnostic Pathology 25 (10.1186/s13000-016-0478-2) 26956509 PMC4784293

[CIT137] Gou WF Sun HZ Zhao S Niu ZF Mao XY Takano Y Zheng HC 2014 Downregulated inhibitor of growth 3 (ING3) expression during colorectal carcinogenesis. Indian Journal of Medical Research 139 561–567. 24927342 PMC4078494

[bib39] Gupta K Gupta S 2017 Neuroendocrine differentiation in prostate cancer: key epigenetic players. Translational Cancer Research S104–S108. (10.21037/tcr.2017.01.20) 30613478 PMC6320222

[bib40] Hanahan D Weinberg RA 2000 The hallmarks of cancer. Cell 57–70. (10.1016/s0092-8674(00)81683-9) 10647931

[bib41] Hanahan D Weinberg RA 2011 Hallmarks of cancer: the next generation. Cell 646–674. (10.1016/j.cell.2011.02.013) 21376230

[bib42] Harris RA Wang T Coarfa C Nagarajan RP Hong C Downey SL Johnson BE Fouse SD Delaney A Zhao Y, 2010 Comparison of sequencing-based methods to profile DNA methylation and identification of monoallelic epigenetic modifications. Nature Biotechnology 1097–1105. (10.1038/nbt.1682) PMC2955169 20852635

[bib43] Hayward SW Wang Y Cao M Hom YK Zhang B Grossfeld GD Sudilovsky D Cunha GR 2001 Malignant transformation in a nontumorigenic human prostatic epithelial cell line. Cancer Research 8135–8142. 11719442

[bib44] He Y Franco OE Jiang M Williams K Love HD Coleman IM Nelson PS Hayward SW 2007 Tissue-specific consequences of cyclin D1 overexpression in prostate cancer progression. Cancer Research 8188–8197. (10.1158/0008-5472.CAN-07-0418) 17804732

[CIT138] Heinecke JL Ridnour LA Cheng RY Switzer CH Lizardo MM Khanna C Glynn SA Hussain SP Young HA Ambs S, *et al* 2014 Tumor microenvironment-based feed-forward regulation of NOS2 in breast cancer progression. PNAS 111 6323–6328. 24733928 10.1073/pnas.1401799111 PMC4035989

[bib45] Hill R Song Y Cardiff RD Van Dyke T 2005 Selective evolution of stromal mesenchyme with p53 loss in response to epithelial tumorigenesis. Cell 1001–1011. (10.1016/j.cell.2005.09.030) 16360031

[bib46] Honeywell RJ Sarkisjan D Kristensen MH de Klerk DJ Peters GJ 2018 DNA methyltransferases expression in normal tissues and various human cancer cell lines, xenografts and tumors. Nucleosides, Nucleotides and Nucleic Acids 696–708. (10.1080/15257770.2018.1498516) 30663502

[bib47] Hu M Yao J Cai L Bachman KE van den Brule F Velculescu V Polyak K 2005 Distinct epigenetic changes in the stromal cells of breast cancers. Nature Genetics 899–905. (10.1038/ng1596) 16007089

[bib48] Huang Y Zhao M Xu H Wang K Fu Z Jiang Y Yao Z 2014 RASAL2 down-regulation in ovarian cancer promotes epithelial-mesenchymal transition and metastasis. Oncotarget 6734–6745. (10.18632/oncotarget.2244) 25216515 PMC4196159

[bib49] Hui K Yue Y Wu S Gu Y Guan B Wang X Hsieh JT Chang LS He D Wu K 2018 The expression and function of RASAL2 in renal cell carcinoma angiogenesis. Cell Death and Disease 881 (10.1038/s41419-018-0898-x) 30158581 PMC6115459

[CIT139] Jeong HM Han J Lee SH Park HJ Lee HJ Choi JS Lee YM Choi YL Shin YK Kwon MJ 2017 ESRP1 is overexpressed in ovarian cancer and promotes switching from mesenchymal to epithelial phenotype in ovarian cancer cells. Oncogenesis 6 e389. 28991261 10.1038/oncsis.2017.87 PMC5668885

[bib50] Jia Z Liu W Gong L Xiao Z 2017 Downregulation of RASAL2 promotes the proliferation, epithelial-mesenchymal transition and metastasis of colorectal cancer cells. Oncology Letters 1379–1385. (10.3892/ol.2017.5581) PMC5403410 28454265

[bib51] Jiang L Gonda TA Gamble MV Salas M Seshan V Tu S Twaddell WS Hegyi P Lazar G Steele I, 2008 Global hypomethylation of genomic DNA in cancer-associated myofibroblasts. Cancer Research 9900–9908. (10.1158/0008-5472.CAN-08-1319) 19047171 PMC2670548

[bib52] Jin B Robertson KD 2013 DNA methyltransferases, DNA damage repair, and cancer. Advances in Experimental Medicine and Biology 3–29. (10.1007/978-1-4419-9967-2_1) PMC3707278 22956494

[bib53] Kalluri R 2016 The biology and function of fibroblasts in cancer. Nature Reviews: Cancer 582–598. (10.1038/nrc.2016.73) 27550820

[CIT140] Kim TW Kang YK Park ZY Kim YH Hong SW Oh SJ Sohn HA Yang SJ Jang YJ Lee DC, *et al* 2014 SH3RF2 functions as an oncogene by mediating PAK4 protein stability. Carcinogenesis 35 624–634. 24130170 10.1093/carcin/bgt338

[bib54] King PD Lubeck BA Lapinski PE 2013 Nonredundant functions for Ras GTPase-activating proteins in tissue homeostasis. Science Signaling re1 (10.1126/scisignal.2003669) 23443682 PMC5483993

[CIT141] Koguchi T Tanikawa C Mori J Kojima Y Matsuda K 2016 Regulation of myo-inositol biosynthesis by p53-ISYNA1 pathway. International Journal of Oncology 48 2415–2424. 27035231 10.3892/ijo.2016.3456

[bib55] Land H Parada LF Weinberg RA 1983 Tumorigenic conversion of primary embryo fibroblasts requires at least two cooperating oncogenes. Nature 596–602. (10.1038/304596a0) 6308472

[CIT142] Langenkamp E Zhang L Lugano R Huang H Elhassan TE Georganaki M Bazzar W Loof J Trendelenburg G Essand M, *et al* 2015 Elevated expression of the C-type lectin CD93 in the glioblastoma vasculature regulates cytoskeletal rearrangements that enhance vessel function and reduce host survival. Cancer Research 75 4504–4516. 26363010 10.1158/0008-5472.CAN-14-3636

[bib56] LeBleu VS Kalluri R 2018 A peek into cancer-associated fibroblasts: origins, functions and translational impact. Disease Models and Mechanisms . (10.1242/dmm.029447) PMC5963854 29686035

[bib58] Lee JS 2007 GSTP1 promoter hypermethylation is an early event in breast carcinogenesis. Virchows Archiv 637–642. (10.1007/s00428-007-0421-8) 17479284

[bib59] Lee WH Morton RA Epstein JI Brooks JD Campbell PA Bova GS Hsieh WS Isaacs WB Nelson WG 1994 Cytidine methylation of regulatory sequences near the pi-class glutathione S-transferase gene accompanies human prostatic carcinogenesis. PNAS 11733–11737. (10.1073/pnas.91.24.11733) 7972132 PMC45306

[bib57] Lee JK Phillips JW Smith BA Park JW Stoyanova T McCaffrey EF Baertsch R Sokolov A Meyerowitz JG Mathis C, 2016 N-Myc drives neuroendocrine prostate cancer initiated from human prostate epithelial cells. Cancer Cell 536–547. (10.1016/j.ccell.2016.03.001) 27050099 PMC4829466

[bib61] Li N Li S 2014 RASAL2 promotes lung cancer metastasis through epithelial-mesenchymal transition. Biochemical and Biophysical Research Communications 358–362. (10.1016/j.bbrc.2014.11.020) 25446096

[bib200] Li H Fan X Kovi RC Jo Y Moquin B Konz R Stoicov C Kurt-Jones E Grossman SR Lyle S, 2007 Spontaneous expression of embryonic factors and p53 point mutations in aged mesenchymal stem cells: a model of age-related tumorigenesis in mice. Cancer Research 10889–10898. (10.1158/0008-5472.CAN-07-2665) 18006834

[bib2001] Li H Wang Y Lu Y Li F 2019 Annexin A2 interacting with ELMO1 regulates HCC chemotaxis and metastasis. Life Sciences 222 168–174. 30853625 10.1016/j.lfs.2019.03.003

[bib63] Lin YH Zhen YY Chien KY Lee IC Lin WC Chen MY Pai LM 2017 LIMCH1 regulates nonmuscle myosin-II activity and suppresses cell migration. Molecular and Cellular Biology 1054–1065. 10.1091/mbc.E15-04-0218 PMC5391182 28228547

[bib631] Liu Q Walker SA Gao D Taylor JA Dai YF Arkell RS Bootman MD Roderick HL Cullen PJ Lockyer PJ 2005 Capri and RASAL impose different modes of information processing on Ras due to contrasting temporal filtering of Ca2+. Journal of Cell Biology 183–190. (10.1083/jcb.200504167) 16009725 PMC1351313

[bib62] Liu D Yang C Bojdani E Murugan AK Xing M 2013 Identification of RASAL1 as a major tumor suppressor gene in thyroid cancer. Journal of the National Cancer Institute 1617–1627. (10.1093/jnci/djt249) 24136889 PMC3818169

[bib64] Lo PK Zhou Q 2018 Emerging techniques in single-cell epigenomics and their applications to cancer research. Journal of Clinical Genomics 1 [epub]. (10.4172/JCG.1000103) PMC6070152 30079405

[CIT144] Lopes BA Meyer C Barbosa TC Zur Stadt U Horstmann M Venn NC Heatley S White DL Sutton R Pombo-de-Oliveira MS, *et al* 2016 COBL is a novel hotspot for IKZF1 deletions in childhood acute lymphoblastic leukemia. Oncotarget 7 53064–53073. 27419633 10.18632/oncotarget.10590 PMC5288169

[bib65] Luczak MW Jagodzinski PP 2006 The role of DNA methylation in cancer development. Folia Histochemica and Cytobiologica 143–154. 16977793

[bib66] Ma BB Sung F Tao Q Poon FF Lui VW Yeo W Chan SL Chan AT 2010 The preclinical activity of the histone deacetylase inhibitor PXD101 (belinostat) in hepatocellular carcinoma cell lines. Investigational New Drugs 107–114. (10.1007/s10637-009-9219-7) 19172229

[bib67] Maeda M Takeshima H Iida N Hattori N Yamashita S Moro H Yasukawa Y Nishiyama K Hashimoto T Sekine S, 2019 Cancer cell niche factors secreted from cancer-associated fibroblast by loss of H3K27me3. Gut [epub]. (10.1136/gutjnl-2018-317645) 31085554

[bib68] McLaughlin SK Olsen SN Dake B De Raedt T Lim E Bronson RT Beroukhim R Polyak K Brown M Kuperwasser C, 2013 The RasGAP gene, RASAL2, is a tumor and metastasis suppressor. Cancer Cell 365–378. (10.1016/j.ccr.2013.08.004) 24029233 PMC3822334

[bib69] Mi S Li Z Chen P He C Cao D Elkahloun A Lu J Pelloso LA Wunderlich M Huang H, 2010 Aberrant overexpression and function of the miR-17-92 cluster in MLL-rearranged acute leukemia. PNAS 3710–3715. (10.1073/pnas.0914900107) 20133587 PMC2840429

[bib70] Minciacchi VR Spinelli C Reis-Sobreiro M Cavallini L You S Zandian M Li X Mishra R Chiarugi P Adam RM, 2017 MYC mediates large oncosome-induced fibroblast reprogramming in prostate cancer. Cancer Research 2306–2317. (10.1158/0008-5472.CAN-16-2942) 28202510

[bib71] Mishra R Haldar S Placencio V Madhav A Rohena-Rivera K Agarwal P Duong F Angara B Tripathi M Liu Z, 2018 Stromal epigenetic alterations drive metabolic and neuroendocrine prostate cancer reprogramming. Journal of Clinical Investigation 4472–4484. (10.1172/JCI99397) 30047926 PMC6159981

[CIT145] Moller-Levet CS Betts GN Harris AL Homer JJ West CM Miller CJ 2009 Exon array analysis of head and neck cancers identifies a hypoxia related splice variant of LAMA3 associated with a poor prognosis. PLoS Computational Biology 5 e1000571. 19936049 10.1371/journal.pcbi.1000571 PMC2773424

[bib72] Moore LD Le T Fan G 2013 DNA methylation and its basic function. Neuropsychopharmacology 23–38. (10.1038/npp.2012.112) 22781841 PMC3521964

[bib73] Muller-Greven G Carlin CR Burgett ME Ahluwalia MS Lauko A Nowacki AS Herting CJ Qadan MA Bredel M Toms SA, 2017 Macropinocytosis of bevacizumab by glioblastoma cells in the perivascular niche affects their survival. Clinical Cancer Research 7059–7071. (10.1158/1078-0432.CCR-17-0249) 28912141 PMC5690835

[CIT146] Mulligan P Westbrook TF Ottinger M Pavlova N Chang B Macia E Shi YJ Barretina J Liu J Howley PM, *et al* 2008 CDYL bridges REST and histone methyltransferases for gene repression and suppression of cellular transformation. Molecular Cell 32 718–726. 19061646 10.1016/j.molcel.2008.10.025 PMC6595072

[bib74] Muro R Nitta T Kitajima M Okada T Suzuki H 2018 Rasal3-mediated T cell survival is essential for inflammatory responses. Biochemical and Biophysical Research Communications 25–30. (10.1016/j.bbrc.2017.12.159) 29291408

[bib75] Nakase I Kobayashi NB Takatani-Nakase T Yoshida T 2015 Active macropinocytosis induction by stimulation of epidermal growth factor receptor and oncogenic Ras expression potentiates cellular uptake efficacy of exosomes. Scientific Reports 10300 (10.1038/srep10300) 26036864 PMC4453128

[bib76] Ohta M Seto M Ijichi H Miyabayashi K Kudo Y Mohri D Asaoka Y Tada M Tanaka Y Ikenoue T, 2009 Decreased expression of the RAS-GTPase activating protein RASAL1 is associated with colorectal tumor progression. Gastroenterology 206–216. (10.1053/j.gastro.2008.09.063) 18992247

[bib78] Okano M Xie S Li E 1998 Cloning and characterization of a family of novel mammalian DNA (cytosine-5) methyltransferases. Nature Genetics 219–220. (10.1038/890) 9662389

[bib77] Okano M Bell DW Haber DA Li E 1999 DNA methyltransferases Dnmt3a and Dnmt3b are essential for de novo methylation and mammalian development. Cell 247–257. (10.1016/s0092-8674(00)81656-6) 10555141

[CIT147] Oliphant MUJ Vincent MY Galbraith MD Pandey A Zaberezhnyy V Rudra P Johnson KR Costello JC Ghosh D DeGregori J, *et al* 2019 SIX2 mediates late-stage metastasis via direct regulation of SOX2 and induction of a cancer stem cell program. Cancer Research 79 720–734. 30606720 10.1158/0008-5472.CAN-18-1791 PMC6586234

[bib79] Olsen SN Wronski A Castano Z Dake B Malone C De Raedt T Enos M DeRose YS Zhou W Guerra S, 2017 Loss of RasGAP tumor suppressors underlies the aggressive nature of luminal B breast cancers. Cancer Discovery 202–217. (10.1158/2159-8290.CD-16-0520) 27974415 PMC6461361

[bib80] Olumi AF Grossfeld GD Hayward SW Carroll PR Tlsty TD Cunha GR 1999 Carcinoma-associated fibroblasts direct tumor progression of initiated human prostatic epithelium. Cancer Research 5002–5011. 10519415 10.1186/bcr138 PMC3300837

[bib81] Otto T Horn S Brockmann M Eilers U Schuttrumpf L Popov N Kenney AM Schulte JH Beijersbergen R Christiansen H, 2009 Stabilization of N-Myc is a critical function of Aurora A in human neuroblastoma. Cancer Cell 67–78. (10.1016/j.ccr.2008.12.005) 19111882

[bib82] Pauken KE Sammons MA Odorizzi PM Manne S Godec J Khan O Drake AM Chen Z Sen DR Kurachi M, 2016 Epigenetic stability of exhausted T cells limits durability of reinvigoration by PD-1 blockade. Science 1160–1165. (10.1126/science.aaf2807) PMC5484795 27789795

[CIT148] Pavlova NN Pallasch C Elia AE Braun CJ Westbrook TF Hemann M Elledge SJ 2013 A role for PVRL4-driven cell-cell interactions in tumorigenesis. eLife 2 e00358. 23682311 10.7554/eLife.00358 PMC3641523

[bib83] Perino M Veenstra GJ 2016 Chromatin control of developmental dynamics and plasticity. Developmental Cell 610–620. (10.1016/j.devcel.2016.08.004) 27676434

[bib84] Pidsley R Lawrence MG Zotenko E Niranjan B Statham A Song J Chabanon RM Qu W Wang H Richards M, 2018 Enduring epigenetic landmarks define the cancer microenvironment. Genome Research 625–638. (10.1101/gr.229070.117) 29650553 PMC5932604

[bib85] Pinto F Pertega-Gomes N Vizcaino JR Andrade RP Carcano FM Reis RM 2016 Brachyury as a potential modulator of androgen receptor activity and a key player in therapy resistance in prostate cancer. Oncotarget 28891–28902. (10.18632/oncotarget.8499) 27049720 PMC5045364

[bib86] Placencio VR Sharif-Afshar AR Li X Huang H Uwamariya C Neilson EG Shen MM Matusik RJ Hayward SW Bhowmick NA 2008 Stromal transforming growth factor-beta signaling mediates prostatic response to androgen ablation by paracrine Wnt activity. Cancer Research 4709–4718. (10.1158/0008-5472.CAN-07-6289) 18559517 PMC2811537

[bib87] Plava J Cihova M Burikova M Matuskova M Kucerova L Miklikova S 2019 Recent advances in understanding tumor stroma-mediated chemoresistance in breast cancer. Molecular Cancer 67 (10.1186/s12943-019-0960-z) 30927930 PMC6441200

[bib88] Qiu W Hu M Sridhar A Opeskin K Fox S Shipitsin M Trivett M Thompson ER Ramakrishna M Gorringe KL, 2008 No evidence of clonal somatic genetic alterations in cancer-associated fibroblasts from human breast and ovarian carcinomas. Nature Genetics 650–655. (10.1038/ng.117) 18408720 PMC3745022

[bib89] Quail DF Joyce JA 2017 The microenvironmental landscape of brain tumors. Cancer Cell 326–341. (10.1016/j.ccell.2017.02.009) 28292436 PMC5424263

[CIT149] Radulovich N Leung L Ibrahimov E Navab R Sakashita S Zhu CQ Kaufman E Lockwood WW Thu KL Fedyshyn Y, *et al* 2015 Coiled-coil domain containing 68 (CCDC68) demonstrates a tumor-suppressive role in pancreatic ductal adenocarcinoma. Oncogene 34 4238–4247. 25381825 10.1038/onc.2014.357 PMC5153324

[bib90] Raffel S Falcone M Kneisel N Hansson J Wang W Lutz C Bullinger L Poschet G Nonnenmacher Y Barnert A, 2017 2017 BCAT1 restricts alphaKG levels in AML stem cells leading to IDHmut-like DNA hypermethylation. Nature 384–388. (10.1038/nature24294) 29144447

[bib91] Raychaudhuri P Park HJ 2011 FoxM1: a master regulator of tumor metastasis. Cancer Research 4329–4333. (10.1158/0008-5472.CAN-11-0640) 21712406 PMC3129416

[bib92] Recouvreux MV Commisso C 2017 Macropinocytosis: a metabolic adaptation to nutrient stress in cancer. Frontiers in Endocrinology 261 (10.3389/fendo.2017.00261) 29085336 PMC5649207

[bib93] Riggs AD Xiong Z 2004 Methylation and epigenetic fidelity. PNAS 4–5. (10.1073/pnas.0307781100) 14695893 PMC314126

[bib94] Robertson KD 2001 DNA methylation, methyltransferases, and cancer. Oncogene 3139–3155. (10.1038/sj.onc.1204341) 11420731

[bib95] Rodriguez-Canales J Hanson JC Tangrea MA Erickson HS Albert PS Wallis BS Richardson AM Pinto PA Linehan WM Gillespie JW, 2007 Identification of a unique epigenetic sub-microenvironment in prostate cancer. Journal of Pathology 410–419. (10.1002/path.2133) 17278115

[bib96] Rose NR Klose RJ 2014 Understanding the relationship between DNA methylation and histone lysine methylation. Biochimica et Biophysica Acta 1362–1372. (10.1016/j.bbagrm.2014.02.007) PMC4316174 24560929

[bib97] Rupaimoole R Calin GA Lopez-Berestein G Sood AK 2016 miRNA deregulation in cancer cells and the tumor microenvironment. Cancer Discovery 235–246. (10.1158/2159-8290.CD-15-0893) 26865249 PMC4783232

[bib98] Saito S Kawamura T Higuchi M Kobayashi T Yoshita-Takahashi M Yamazaki M Abe M Sakimura K Kanda Y Kawamura H, 2015 RASAL3, a novel hematopoietic RasGAP protein, regulates the number and functions of NKT cells. European Journal of Immunology 1512–1523. (10.1002/eji.201444977) 25652366

[CIT150] Sandsmark E Andersen MK Bofin AM Bertilsson H Drablos F Bathen TF Rye MB Tessem MB 2017 SFRP4 gene expression is increased in aggressive prostate cancer. Scientific Reports 7 14276. 29079735 10.1038/s41598-017-14622-3 PMC5660209

[bib99] Scheffzek K Shivalingaiah G 2019 Ras-specific GTPase-activating proteins-structures, mechanisms, and interactions. Cold Spring Harbor Perspectives in Medicine a031500 (10.1101/cshperspect.a031500) 30104198 PMC6396337

[bib100] Schmechel D Marangos PJ Brightman M 1978 Neurone-specific enolase is a molecular marker for peripheral and central neuroendocrine cells. Nature 834–836. (10.1038/276834a0) 31568

[CIT151] Schroder B 2016 The multifaceted roles of the invariant chain CD74–more than just a chaperone. Biochimica et Biophysica Acta 1863 1269–1281. 27033518 10.1016/j.bbamcr.2016.03.026

[bib101] Sen DR Kaminski J Barnitz RA Kurachi M Gerdemann U Yates KB Tsao HW Godec J LaFleur MW Brown FD, 2016 The epigenetic landscape of T cell exhaustion. Science 1165–1169. (10.1126/science.aae0491) 27789799 PMC5497589

[bib102] Simanshu DK Nissley DV McCormick F 2017 RAS proteins and their regulators in human disease. Cell 17–33. (10.1016/j.cell.2017.06.009) PMC5555610 28666118

[bib103] Smith B Agarwal P Bhowmick NA 2017 MicroRNA applications for prostate, ovarian and breast cancer in the era of precision medicine. Endocrine-Related Cancer R157–R172. (10.1530/ERC-16-0525) 28289080 PMC5446589

[bib104] Smits M van Rijn S Hulleman E Biesmans D van Vuurden DG Kool M Haberler C Aronica E Vandertop WP Noske DP, 2012 EZH2-regulated DAB2IP is a medulloblastoma tumor suppressor and a positive marker for survival. Clinical Cancer Research 4048–4058. (10.1158/1078-0432.CCR-12-0399) 22696229

[CIT152] Song Y Fu LJ Li HT Qiu XG 2019 Evaluation of MEDAG gene expression in papillary thyroid microcarcinoma: associations with histological features, regional lymph node metastasis and prognosis. Scientific Reports 9 5800. 30967566 10.1038/s41598-019-41701-4 PMC6456583

[bib105] Sproul D Meehan RR 2013 Genomic insights into cancer-associated aberrant CpG island hypermethylation. Briefings in Functional Genomics 174–190. (10.1093/bfgp/els063) 23341493 PMC3662888

[bib106] Stefanska B Cheishvili D Suderman M Arakelian A Huang J Hallett M Han ZG Al-Mahtab M Akbar SM Khan WA, 2014 Genome-wide study of hypomethylated and induced genes in patients with liver cancer unravels novel anticancer targets. Clinical Cancer Research 3118–3132. (10.1158/1078-0432.CCR-13-0283) 24763612

[bib107] Still ER Yuneva MO 2017 Hopefully devoted to Q: targeting glutamine addiction in cancer. British Journal of Cancer 1375–1381. (10.1038/bjc.2017.113) 28441384 PMC5520092

[CIT153] Sun B Hou YL Hou WR Zhang SN Ding X Su XL 2012 cDNA cloning, overexpression, purification and pharmacologic evaluation for anticancer activity of ribosomal protein L23A gene (RPL23A) from the Giant Panda. International Journal of Molecular Sciences 13 2133–2147. 22408443 10.3390/ijms13022133 PMC3292012

[CIT154] Takayama KI Suzuki T Fujimura T Takahashi S Inoue S 2018 COBLL1 modulates cell morphology and facilitates androgen receptor genomic binding in advanced prostate cancer. PNAS 115 4975–4980. 29686105 10.1073/pnas.1721957115 PMC5948986

[bib108] Takebayashi S Tamura T Matsuoka C Okano M 2007 Major and essential role for the DNA methylation mark in mouse embryogenesis and stable association of DNMT1 with newly replicated regions. Molecular and Cellular Biology 8243–8258. (10.1128/MCB.00899-07) 17893328 PMC2169176

[bib109] Tan HL Sood A Rahimi HA Wang W Gupta N Hicks J Mosier S Gocke CD Epstein JI Netto GJ, 2014 Rb loss is characteristic of prostatic small cell neuroendocrine carcinoma. Clinical Cancer Research 890–903. (10.1158/1078-0432.CCR-13-1982) 24323898 PMC3931005

[CIT155] Teo WW Merino VF Cho S Korangath P Liang X Wu RC Neumann NM Ewald AJ Sukumar S 2016 HOXA5 determines cell fate transition and impedes tumor initiation and progression in breast cancer through regulation of E-cadherin and CD24. Oncogene 35 5539–5551. 27157614 10.1038/onc.2016.95 PMC5073039

[bib131] Teufel A Weinmann A Galle PR Lohse AW 2005 *In silico* characterization of LZTS3, a potential tumor suppressor. Oncology Reports 547–551. 16012743

[bib110] Tirosh I Barkai N 2008 Two strategies for gene regulation by promoter nucleosomes. Genome Research 1084–1091. (10.1101/gr.076059.108) 18448704 PMC2493397

[bib111] Trimboli AJ Cantemir-Stone CZ Li F Wallace JA Merchant A Creasap N Thompson JC Caserta E Wang H Chong JL, 2009 Pten in stromal fibroblasts suppresses mammary epithelial tumours. Nature 1084–1091. (10.1038/nature08486) PMC2767301 19847259

[bib112] Tyan SW Hsu CH Peng KL Chen CC Kuo WH Lee EY Shew JY Chang KJ Juan LJ Lee WH 2012 Breast cancer cells induce stromal fibroblasts to secrete ADAMTS1 for cancer invasion through an epigenetic change. PLoS ONE e35128 (10.1371/journal.pone.0035128) 22514714 PMC3325931

[CIT156] Vaishnavi A Capelletti M Le AT Kako S Butaney M Ercan D Mahale S Davies KD Aisner DL Pilling AB, *et al* 2013 Oncogenic and drug-sensitive NTRK1 rearrangements in lung cancer. Nature Medicine 19 1469–1472. (10.1038/nm.3352) PMC3823836 24162815

[bib113] Valencia T Kim JY Abu-Baker S Moscat-Pardos J Ahn CS Reina-Campos M Duran A Castilla EA Metallo CM Diaz-Meco MT, 2014 Metabolic reprogramming of stromal fibroblasts through p62-mTORC1 signaling promotes inflammation and tumorigenesis. Cancer Cell 121–135. (10.1016/j.ccr.2014.05.004) 25002027 PMC4101061

[bib114] Vizoso M Puig M Carmona FJ Maqueda M Velasquez A Gomez A Labernadie A Lugo R Gabasa M Rigat-Brugarolas LG, 2015 Aberrant DNA methylation in non-small cell lung cancer-associated fibroblasts. Carcinogenesis 1453–1463. (10.1093/carcin/bgv146) 26449251 PMC4662832

[CIT157] Wang CA Drasin D Pham C Jedlicka P Zaberezhnyy V Guney M Li H Nemenoff R Costello JC Tan AC, *et al* 2014 Homeoprotein Six2 promotes breast cancer metastasis via transcriptional and epigenetic control of E-cadherin expression. Cancer Research 74 7357–7370. 25348955 10.1158/0008-5472.CAN-14-0666 PMC4268359

[bib116] Wang Z Wang J Su Y Zeng Z 2015 RASAL2 inhibited the proliferation and metastasis capability of nasopharyngeal carcinoma. International Journal of Clinical and Experimental Medicine 18765–18771. 26770493 PMC4694393

[bib117] Wang ZR Wei JH Zhou JC Haddad A Zhao LY Kapur P Wu KJ Wang B Yu YH Liao B, 2016 Validation of DAB2IP methylation and its relative significance in predicting outcome in renal cell carcinoma. Oncotarget 31508–31519. (10.18632/oncotarget.8971) 27129174 PMC5058774

[bib115] Wang X Sheng W Wang Y Li L Li Y Zhang S Liu X Chen S Zhen Y 2018 A macropinocytosis-intensifying albumin domain-based scFv antibody and its conjugate directed against K-Ras mutant pancreatic cancer. Molecular Pharmaceutics 2403–2412. (10.1021/acs.molpharmaceut.8b00234) 29757658

[bib118] Wiedenmann B Franke WW Kuhn C Moll R Gould VE 1986 Synaptophysin: a marker protein for neuroendocrine cells and neoplasms. PNAS 3500–3504. (10.1073/pnas.83.10.3500) 3010302 PMC323544

[bib120] Wu Y Garmire LX Fan R 2012 Inter-cellular signaling network reveals a mechanistic transition in tumor microenvironment. Integrative Biology 1478–1486. (10.1039/c2ib20044a) 23080410 PMC3502715

[bib119] Wu J Gu Y Xiao Y Xia C Li H Kang Y Sun J Shao Z Lin Z Zhao X 2018 Characterization of DNA methylation associated gene regulatory networks during stomach cancer progression. Frontiers in Genetics 711 (10.3389/fgene.2018.00711) 30778372 PMC6369581

[bib121] Xiao M Yang H Xu W Ma S Lin H Zhu H Liu L Liu Y Yang C Xu Y, 2012 Inhibition of alpha-KG-dependent histone and DNA demethylases by fumarate and succinate that are accumulated in mutations of FH and SDH tumor suppressors. Genes and Development 1326–1338. (10.1101/gad.191056.112) 22677546 PMC3387660

[bib122] Xiao Q Zhou D Rucki AA Williams J Zhou J Mo G Murphy A Fujiwara K Kleponis J Salman B, 2016 Cancer-associated fibroblasts in pancreatic cancer are reprogrammed by tumor-induced alterations in genomic DNA methylation. Cancer Research 5395–5404. (10.1158/0008-5472.CAN-15-3264) 27496707 PMC5026619

[CIT158] Xing D Wang J Ou S Wang Y Qiu B Ding D Guo F Gao Q 2014 Expression of neonatal Nav1.5 in human brain astrocytoma and its effect on proliferation, invasion and apoptosis of astrocytoma cells. Oncology Reports 31 2692–2700. 24756536 10.3892/or.2014.3143

[CIT159] Xu X Tan X Tampe B Nyamsuren G Liu X Maier LS Sossalla S Kalluri R Zeisberg M Hasenfuss G, *et al* 2015 Epigenetic balance of aberrant Rasal1 promoter methylation and hydroxymethylation regulates cardiac fibrosis. Cardiovascular Research 105 279–291. 25616414 10.1093/cvr/cvv015

[bib123] Yadav KK Shameer K Readhead B Yadav SS Li L Kasarskis A Tewari AK Dudley JT 2016 Systems medicine approaches to improving understanding, treatment, and clinical management of neuroendocrine prostate cancer. Current Pharmaceutical Design 5234–5248. (10.2174/1381612822666160513145924) 27174811

[bib124] Yan L Zhou J Gao Y Ghazal S Lu L Bellone S Yang Y Liu N Zhao X Santin AD, 2015 Regulation of tumor cell migration and invasion by the H19/let-7 axis is antagonized by metformin-induced DNA methylation. Oncogene 3076–3084. (10.1038/onc.2014.236) 25088204

[bib125] Yan M Li X Tong D Han C Zhao R He Y Jin X 2016 miR-136 suppresses tumor invasion and metastasis by targeting RASAL2 in triple-negative breast cancer. Oncology Reports 65–71. (10.3892/or.2016.4767) 27108696 PMC4899014

[CIT160] Yang X Duan B Zhou X 2017 Long non-coding RNA FOXD2-AS1 functions as a tumor promoter in colorectal cancer by regulating EMT and Notch signaling pathway. European Review for Medical and Pharmacological Sciences 21 3586–3591. 28925486

[bib126] Yano M Toyooka S Tsukuda K Dote H Ouchida M Hanabata T Aoe M Date H Gazdar AF Shimizu N 2005 Aberrant promoter methylation of human DAB2 interactive protein (hDAB2IP) gene in lung cancers. International Journal of Cancer 59–66. (10.1002/ijc.20531) 15386433

[bib127] Yu J Walter K Omura N Hong SM Young A Li A Vincent A Goggins M 2012 Unlike pancreatic cancer cells pancreatic cancer associated fibroblasts display minimal gene induction after 5-aza-2′-deoxycytidine. PLoS ONE e43456 (10.1371/journal.pone.0043456) 22984426 PMC3439436

[CIT161] Yuan H Kajiyama H Ito S Yoshikawa N Hyodo T Asano E Hasegawa H Maeda M Shibata K Hamaguchi M, *et al* 2013 ALX1 induces snail expression to promote epithelial-to-mesenchymal transition and invasion of ovarian cancer cells. Cancer Research 73 1581–1590. 23288509 10.1158/0008-5472.CAN-12-2377

[CIT162] Zhai LL Xie Q Zhou CH Huang DW Tang ZG Ju TF 2017 Overexpressed HSPA2 correlates with tumor angiogenesis and unfavorable prognosis in pancreatic carcinoma. Pancreatology 17 457–463. 28416384 10.1016/j.pan.2017.04.007

[bib128] Zhang B Pan X Cobb GP Anderson TA 2007 MicroRNAs as oncogenes and tumor suppressors. Developmental Biology 1–12. (10.1016/j.ydbio.2006.08.028) 16989803

[CIT163] Zhang H Wang Y Liu Z Yao B Dou C Xu M Li Q Jia Y Wu S Tu K, *et al* 2016 Lymphocyte-specific protein 1 inhibits the growth of hepatocellular carcinoma by suppressing ERK1/2 phosphorylation. FEBS Open Bio 6 1227–1237. 10.1002/2211-5463.12139 PMC5324767 28255535

[CIT164] Zhang JY Zhang PP Zhou WP Yu JY Yao ZH Chu JF Yao SN Wang C Lone W Xia QX, *et al* 2019 L-type cav 1.2 calcium channel-alpha-1C regulates response to rituximab in diffuse large B-cell lymphoma. Clinical Cancer Research 25 4168–4178. 30824586 10.1158/1078-0432.CCR-18-2146 PMC9161643

[CIT165] Zong M Meng M Li L 2011 Low expression of TBX4 predicts poor prognosis in patients with stage II pancreatic ductal adenocarcinoma. International Journal of Molecular Sciences 12 4953–4963. 21954337 10.3390/ijms12084953 PMC3179144

[bib129] Zong Y Huang J Sankarasharma D Morikawa T Fukayama M Epstein JI Chada KK Witte ON 2012 Stromal epigenetic dysregulation is sufficient to initiate mouse prostate cancer via paracrine Wnt signaling. PNAS E3395–E3404. (10.1073/pnas.1217982109) 23184966 PMC3528570

[bib130] Zwartkruis FJ Burgering BM 2013 Ras and macropinocytosis: trick and treat. Cell Research 982–983. (10.1038/cr.2013.79) 23774265 PMC3731563

